# Dr. Joseph Robinson

**Published:** 1873-10

**Authors:** 


JOSEPH ROBINSON, D. D. S.—It is with regret that we announce the death of Dr. Joseph Robinson, a graduate of the Baltimore College of Dental Surgery, of the Class of 1865, which occurred near Black Horse, Maryland, August 31st, 1873.

Dr. Robinson practiced dentistry in Baltimore from the time he graduated until a few months prior to his death, when failing health compelled him to leave the city for his country home. Dr. Robinson was of a modest and retiring disposition, and was noted for his upright, gentlemanly conduct.

His desire appeared to be to practice his profession in such a manner that no act of his should in any way detract from its respectability and usefulness; and his mind was imbued with correct ideas in regard to professional courtesy and justice.

